# The effect of delaying initiation with umeclidinium/vilanterol in patients with COPD: an observational administrative claims database analysis using marginal structural models

**DOI:** 10.1186/s40248-018-0151-6

**Published:** 2018-10-11

**Authors:** Ami R. Buikema, Lee Brekke, Amy Anderson, Eleena Koep, Damon Van Voorhis, Lucie Sharpsten, Beth Hahn, Riju Ray, Richard H. Stanford

**Affiliations:** 1 Health Economics and Outcomes Research, Optum, 11000 Optum Circle, Eden Prairie, MN 55344 USA; 2 US Value Evidence and Outcomes, GSK, 5 Moore Drive, Research Triangle Park, NC 27709-3398 USA; 3 US Medical Affairs, GSK, 5 Moore Drive, Research Triangle Park, NC 27709-3398 USA

**Keywords:** COPD, Umeclidinium/vilanterol, Exacerbation, Costs, Time-varying confounding, Marginal structural model

## Abstract

**Background:**

Chronic obstructive pulmonary disease (COPD) is associated with high clinical and economic burden. Optimal pharmacological therapy for COPD aims to reduce symptoms and the frequency and severity of exacerbations. Umeclidinium/vilanterol (UMEC/VI) is an approved combination therapy for once-daily maintenance treatment of patients with COPD. This study evaluated the impact of delaying UMEC/VI initiation on medical costs and exacerbation risk.

**Methods:**

A retrospective analysis of patients with COPD who initiated UMEC/VI between 4/28/2014 and 7/31/2016 was conducted using the Optum Research Database. The index date was the first COPD visit after UMEC/VI available on US formulary (Commercial 4/28/2014; Medicare Advantage 1/1/2015). Patients were followed for 12 months post-index, and categorized into 12 cohorts corresponding to month (30-day period) of UMEC/VI initiation (i.e. Months 1–12) post-index. The outcomes studied during the follow up period included COPD-related and all-cause medical costs, and risk of COPD exacerbations. Marginal structural models (MSM) were used to control for time-varying confounding due to changes in treatment and severity during follow up.

**Results:**

2,200 patients initiating UMEC/VI were included in the study sample. Patients’ average age was 69.3 years, 49.9% were female and 69.7% were Medicare insured. Following MSM analysis, 12-month adjusted COPD-related medical costs increased by 2.9% (95% confidence interval [CI]: 0.1–5.9%; *p* = 0.044) for each monthly delay in UMEC/VI initiation, with a 37.4% higher adjusted cost for patients initiating UMEC/VI in Month 12 versus Month 1 ($13,087 vs. $9524). The 12-month adjusted all-cause medical costs increased by 2.8% (95% CI: 0.6–5.2%; *p* = 0.013) for each monthly delay, with a 36.1% higher adjusted cost for patients initiating UMEC/VI at Month 12 versus Month 1 ($22,766 vs. $16,727). The monthly risk of severe exacerbation was significantly higher in patients who had not yet initiated UMEC/VI than those who had (hazard ratio: 1.74; 95% CI: 1.35–2.23; *p* < 0.001).

**Conclusions:**

Prompt use of UMEC/VI following a physician visit for COPD appears to result in economic and clinical benefits, with reductions in medical costs and exacerbation risk. Additional research is warranted to assess the benefits of initiating UMEC/VI as a first-line therapy compared with escalation to UMEC/VI from monotherapies.

**Electronic supplementary material:**

The online version of this article (10.1186/s40248-018-0151-6) contains supplementary material, which is available to authorized users.

## Background

Chronic obstructive pulmonary disease (COPD) is a chronic and disabling respiratory disease characterized by airflow obstruction and persistent breathing difficulties [1]. In the United States (US), COPD is among the top 3 causes of death [2], and, in 2013, was reported to affect 15.7 million people (6.4% of the population) [3]. COPD has an unprecedented economic burden on patients and the healthcare system. Almost $4 billion in absenteeism costs due to COPD were reported in the US in 2010, and direct medical costs associated with COPD are estimated to reach nearly $50 billion by 2020 [4]. The most important factors contributing to the financial burden of COPD on society are disease severity, the presence of frequent exacerbations, and the presence of comorbidities, which are common [5]. Up to 75% of COPD costs are attributable to exacerbations [6], and they are the most frequent reason for hospital admission [7]. Treatments and therapies that can reduce direct medical services, such as emergency department (ED) visits and hospitalizations, may help to reduce the medical costs associated with COPD.

Although COPD is a progressive, irreversible disease, pharmacological treatment can help to control symptoms (such as chronic cough, chronic sputum production, and dyspnea), improve lung function, and reduce exacerbations, as well as improve exercise tolerance and health status [1, 8]. Treatment decisions are guided by the Global initiative for chronic Obstructive Lung Disease (GOLD) report and reflect that COPD is not a static disease, with treatment options usually requiring updates and enhancements as the patient’s status changes [1, 9].

The mainstay of pharmacological therapy for COPD is bronchodilation with a long-acting muscarinic antagonist (LAMA), a long-acting β_2_-agonist (LABA), or a combination of the two, depending on the symptom burden and exacerbation history [1, 8, 10, 11]. Currently, for symptomatic patients with low exacerbation risk, LABA or LAMA monotherapy is recommended as initial therapy; for patients with persistent symptoms or further exacerbations on monotherapy, the use of two bronchodilators should be considered [1]. Patients with persistent exacerbations may also benefit from dual LABA/LAMA therapy or the combination of a LABA with an inhaled corticosteroid (ICS) [1]. Patients who develop further exacerbations or experience further symptoms on LABA/LAMA or ICS/LABA therapy can also escalate treatment to triple therapy with a LABA/LAMA/ICS combination [1].

Umeclidinium/vilanterol 62.5/25 mcg (UMEC/VI) is a once-daily single inhaler combination LAMA/LABA that was approved by the US Food and Drug Administration (FDA) in December 2013 for the treatment of COPD [12]. In clinical trials, UMEC/VI consistently demonstrated improvements in lung function in symptomatic patients with moderate-to-severe COPD and low risk of exacerbation when compared with placebo [13, 14], UMEC or VI monotherapy [15, 16], or tiotropium [16–18]. Lung function improvements have also been demonstrated against the ICS/LABA fluticasone propionate/salmeterol [19]. To date, there are no studies assessing the effects of earlier initiation of a LAMA/LABA combination therapy versus delayed initiation of dual therapy on clinical outcomes and long-term COPD-related costs.

This study aims to investigate the effect of delaying UMEC/VI on 12-month COPD-related medical costs, 12-month all-cause medical costs, COPD exacerbation event rates, and exacerbation risk.

## Methods

### Study design

**Fig. 1:**
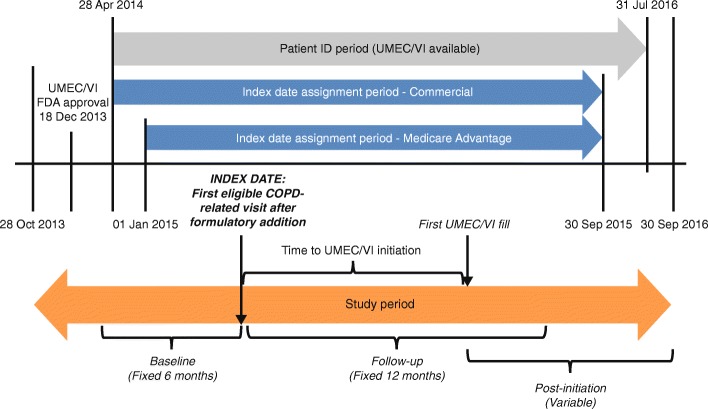
Study design. COPD, chronic obstructive pulmonary disease; FDA, Food and Drug Administration; ID, identification; UMEC/VI, umeclidinium/vilanterol

This was a retrospective observational administrative claims database analysis of patients enrolled in commercial and Medicare Advantage (MA) with part D (MAPD) healthcare plans initiating treatment with UMEC/VI (study number 206409 [HO-16-16,346]). The patient identification [ID] period ran from April 28, 2014 (first commercial access of UMEC/VI in the US) to July 31, 2016 (the most recent date when fully adjudicated pharmacy information was available for patients within the database) to identify patients treated with UMEC/VI (Fig. 1). The index date for each patient was defined as the date of the first eligible COPD-related visit with a potential prescriber of UMEC/VI after formulary addition (April 28, 2014 for commercial enrollees and January 01, 2015 for MAPD enrollees (Fig. 1)) through September 30, 2015 (index date assignment period). Potential prescribers included general practitioners, internists, pulmonologists, cardiologists, allergists, ear, nose and throat specialists, obstetricians/gynecologists, and emergency medicine specialists. Patients were observed for 6 months prior to the index date for measurement of baseline covariates and for 12 months following the index date to assess outcomes. Patients were assigned to 12 cohorts corresponding to each month (30 days) following index when UMEC/VI was initiated (Months 1–12).

### Data sources

Patient data were obtained from the Optum Research Database (ORD), which contains medical data, pharmacy data, and enrollment information for a geographically diverse and representative patient population across the US (approximately 19% of the US population in commercial health plans and 17% of those in MA plans were represented in 2016).

### Patients

Eligible patients were ≥ 40 years old in the index year with ≥1 prescription fill for UMEC/VI during the patient ID period and ≥ 1 COPD-related visit with a potential prescriber during the index date assignment period. Visits were considered COPD-related if they had a diagnosis code for COPD (International Classification of Disease, 9^th^ edition, clinical modification [ICD-9-CM] codes: 491.xx, 492.x, 493.2×, 496 or International Classification of Disease, 10^th^ edition, clinical modification [ICD-10-CM] codes: J41*, J42, J43*, J44*) in any position on the visit claim. Patients must also have had ≥1 prescription fill for UMEC/VI within 12 months following the index date. Continuous enrollment with both medical and pharmacy coverage from the start of formulary availability to the index date, for ≥6 months prior to the index date, and for ≥12 months on and following the index date was required. Patients were excluded if they had used UMEC/VI or any other LAMA/LABA fixed dose combination therapy during the 6-month baseline period.

### Measures

Patient demographics were captured from enrollment records. Economic and clinical characteristics were assessed during the 6-month baseline period. Outcomes were measured in the 12-month follow up period.

Cost outcomes were computed as combined health plan and patient-paid amounts from medical claims during the 12 months following (and including) the index date. Medical costs were considered attributable to COPD if the claim had a diagnosis code for COPD in any position. COPD-attributable pharmacy costs were identified by any pharmacy claim for a COPD-related treatment (Additional file 1). Both COPD-related and all-cause healthcare costs were adjusted to 2015 dollars (USD [$]) using the annual medical care component of the Consumer Price Index to reflect inflation [20]; costs included combined health plan and patient-paid amounts.

The COPD-related exacerbations outcome was captured in the follow up period, not including the index date. A severe COPD exacerbation was defined as a hospitalization with a diagnosis code for COPD in any position. A moderate exacerbation was defined as a COPD-related ED, physician office, or urgent care visit with a prescription for a systemic or oral corticosteroid, or a COPD guideline-recommended antibiotic, within 5 days. Exacerbations occurring within 14 days of each other were considered to be a single exacerbation episode, and were classified according to the highest severity contributing event. For the longitudinal marginal structural model (MSM) analyses, exacerbations were captured in monthly indicators during the 12 months following the index date.

### Statistical analyses

Multivariable analyses were conducted using MSMs [21, 22] to evaluate the impact of the month of UMEC/VI treatment initiation on follow up cost outcomes and COPD exacerbation incidence. A two-step process was used: 1) each subject’s probability of having their own treatment history was estimated to derive inverse probability weights, which were subsequently stabilized using the procedures recommended in the literature [21, 22]. Logistic treatment selection models with pooled monthly time periods were used to calculate the stabilized weights; then 2) weighted generalized estimating equation (GEE) models were used to evaluate the cost and exacerbation incidence outcomes.

Treatment selection weighting models for the cost analyses contained potential confounders as independent variables, including severity indicators such as exacerbations, rescue medication prescription fills, respiratory infections, emphysema and Charlson comorbidity index. The complete list of variables used in the weighting models are described in Additional file 2.

The 12-month COPD-related and all-cause medical cost outcome analyses were estimated for one observation per subject, using weighted GEE with a gamma distribution, a log link, and sandwich variance estimation. The covariates included the primary exposure of interest, month of treatment initiation, as well as the baseline variables used in the weighting models. The analysis was performed using the same weighting scheme for COPD-related and all-cause medical cost outcomes, with weights truncated at the 99.9^th^ percentile [23]. The risk of first severe exacerbation was modeled for one observation per subject per month using GEE weighted repeated measures [24], GEE with a logit link, and sandwich variance estimator. The exposure comprised two key terms: 1) a variable indicating if a patient was not receiving UMEC/VI in a given month (an “untreated” flag); and 2) an “untreated” interaction term with month (centered at 6 months). Additional covariates included the baseline variables used in the weighting models. Weights were truncated at the 99.9^th^ percentile. If patients had an exacerbation and initiated treatment in the same month, and the exacerbation occurred prior to treatment initiation, treatment initiation was assigned to the following month. All analyses were conducted using SAS version 9.4 (SAS Institute, Cary, NC, USA).

### Sensitivity analyses

Sensitivity analyses were conducted to examine the impact of various modifications to the MSM assumptions and covariates on the outcomes. Details of the sensitivity analyses are presented in Additional file 2.

## Results

### Patient demographics

**Fig. 2:**
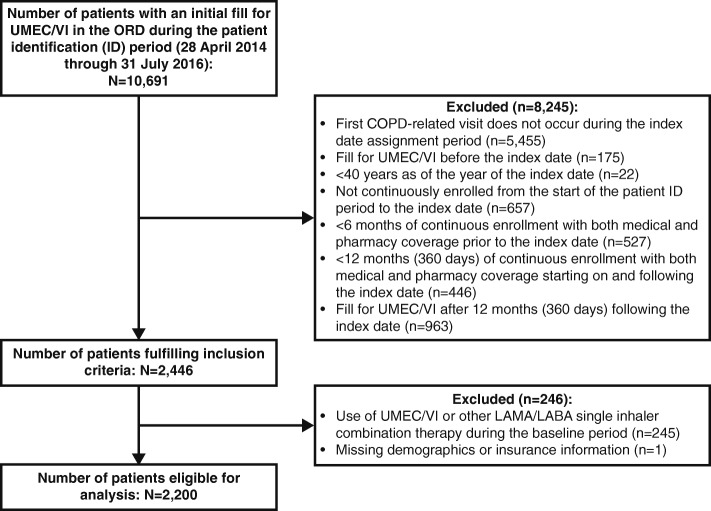
Patient disposition. COPD, chronic obstructive pulmonary disease; LABA, long-acting β_2_-agonist; LAMA, long-acting muscarinic antagonist; ORD, Optum Research Database; UMEC/VI, umeclidinium/vilanterol

A total of 8,317,017 patients aged 40 years and older (6,253,949 patients enrolled in commercial plans and 2,063,068 in MAPD) had at least one day of enrollment during the index date assignment periods. Out of these, a total of 10,691 patients initiated treatment with UMEC/VI and 2,200 patients met all eligibility criteria and were included in the analytic population (Fig. 2). The mean (standard deviation [SD]) age of the population was 69.3 (9.9) years and 69.7% of patients were enrolled in MAPD plans. Approximately half of the patients (49.9%) were female and the mean (SD) Charlson comorbidity score was 1.5 (1.5). Hypertension was a common comorbid condition (present in 64.4% of patients) and asthma was present in 17.3% of patients (Table 1). More than one-fifth (22.3%) of patients initiated treatment with UMEC/VI within Month 1; and 60.3% initiated treatment by Month 6. After Month 3 post-index, the number of patients initiating treatment in each month was relatively similar, averaging 142 patients per month (Additional file 3).

**Table 1:** Baseline characteristics

Characteristic	Total patients (*N* = 2,200)
Mean age, mean (SD), years	69.3 (9.9)
Age category, n (%), years	
40–59	414 (18.8)
60–64	268 (12.2)
65–74	824 (37.5)
75–84	576 (26.2)
≥ 85	118 (5.4)
Female, n (%)	1,098 (49.9)
Insurance type, n (%)	
Commercial	666 (30.3)
Medicare Advantage	1,534 (69.7)
Charlson comorbidity score, mean (SD)	1.5 (1.5)
Baseline (6 month) COPD exacerbations, mean (SD)	0.7 (0.9)
Severe	0.1 (0.4)
Moderate	0.6 (0.8)
Comorbidities, n (%)	
COPD and bronchiectasis	2,154 (97.9)
Hypertension	1,417 (64.4)
Other lower respiratory disease	1,353 (61.5)
Disorders of lipid metabolism	1,215 (55.2)
Asthma	380 (17.3)
Baseline maintenance therapy use, n (%)	
LAMA	624 (28.4)
ICS	138 (6.3)
ICS/LABA	584 (26.6)
Baseline respiratory medication use, n (%)	
OCS	836 (38.0)
SABA	899 (40.9)
SAMA	65 (3.0)
SAMA/SABA	252 (11.5)

*COPD* chronic obstructive pulmonary disease, *CPI* consumer price index, *ICS* inhaled corticosteroid, *LABA* long-acting β_2_-agonist, *LAMA* long-acting muscarinic antagonist, *OCS* oral corticosteroid, *SABA* short-acting β_2_-agonist, *SAMA* short-acting muscarinic antagonist, *SD* standard deviation

### COPD-related medical costs

**Fig. 3:**
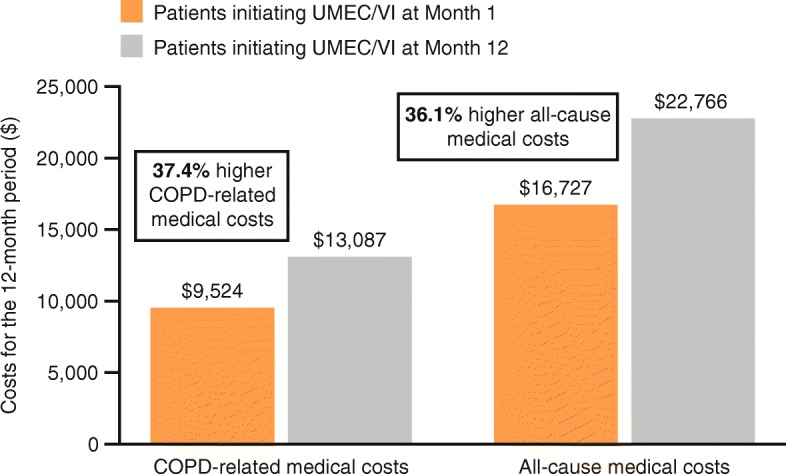
12-month adjusted* COPD-related and all-cause medical costs comparing UMEC/VI initiation at Months 1 and 12. *Adjusted for baseline and time-varying covariates, including: demographics, comorbidities, exacerbation, treatment and utilization. Month of delay in UMEC/VI treatment initiation: *p* = 0.044 for COPD-related medical costs; *p* = 0.013 for all-cause medical costs. COPD, chronic obstructive pulmonary disease; UMEC/VI, umeclidinium/vilanterol

Based on MSM analysis with weights truncated at the 99.9^th^ percentile, the adjusted COPD-related medical costs during the 12-month post-index period were 2.9% higher (95% confidence interval [CI]: 0.1–5.9%; *p* = 0.044) for each month of delay in initiation of UMEC/VI. Patients initiating UMEC/VI therapy in Month 1 had a 12-month adjusted cost of $9,524 versus $13,087 for those initiating UMEC/VI therapy in Month 12. Overall, these results demonstrated that patients initiating treatment with UMEC/VI in Month 12 had 37.4% higher COPD-related medical costs than patients initiating within Month 1 (Fig. 3).

Sensitivity analyses were conducted to examine the impact of various modifications to the MSM assumptions and covariates on the outcomes. Details of the sensitivity analyses are presented in Additional file 4.

### All-cause medical costs

Based on MSM analysis with weights truncated at the 99.9^th^ percentile, adjusted all-cause medical costs were estimated to increase by 2.8% (95% CI: 0.6–5.2%; *p* = 0.013) for each month of delay in initiation of UMEC/VI. Patients who initiated UMEC/VI in Month 1 had first-year adjusted costs of $16,727 compared with $22,766 for patients who initiated UMEC/VI in Month 12. Overall, these results demonstrated that patients initiating treatment with UMEC/VI in Month 12 had 36.1% higher all-cause medical costs than patients initiating treatment within Month 1 (Fig. 3).

### COPD exacerbation event rates and risks associated with delay in UMEC/VI initiation

Overall, 63.3% of patients in the study population experienced a COPD exacerbation during the follow up period, with 21.2% of patients having at least one severe exacerbation. The mean (SD) number of exacerbations during the follow up period was 1.4 (1.7), while the mean (SD) numbers of moderate and severe exacerbations were 1.1 (1.4) and 0.3 (0.7), respectively.

**Fig. 4:**
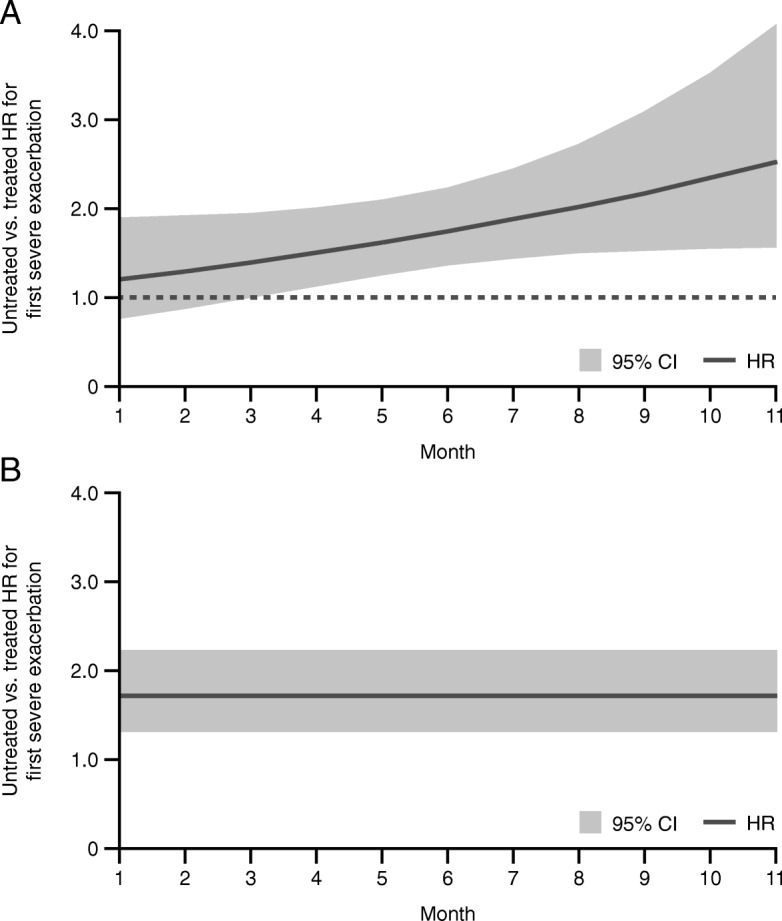
Comparison of HR by UMEC/VI initiation month (**a**) with and (**b**) without time interaction term. CI, confidence interval; HR, hazard ratio; UMEC/VI, umeclidinium/vilanterol

Based on MSM analysis, the risk of experiencing a severe exacerbation was significantly higher in patients who had not yet initiated UMEC/VI compared with those who had (hazard ratio [HR] for term 1: 1.74; 95% CI: 1.35–2.23; *p* < 0.001; HR for the untreated/month interaction, term 2: 1.08; 95% CI: 1.00–1.17; *p* = 0.067; combined *p*-value *p* < 0.001). Inclusion of the time interaction term in the final model demonstrated a 20% increased risk of a first severe exacerbation for patients not receiving UMEC/VI in the first 30 days relative to patients receiving UMEC/VI in the first 30 days post-index; for each additional month of treatment delay, the risk of a first severe exacerbation increased by 8% (Fig. 4a). Without inclusion of the time interaction term in the model, patients who had not yet initiated UMEC/VI had a 70% increased risk of first severe exacerbation in every month that they remained untreated compared with patients who initiated treatment with UMEC/VI in the current or prior months (HR: 1.70; 95% CI: 1.30–2.23; *p* < 0.001) (Fig. 4b).

## Discussion

This large, retrospective observational cohort study has demonstrated that, among patients initiating treatment with the LAMA/LABA therapy, UMEC/VI, within a year of their first eligible COPD-related visit, consistently lower COPD-related and all-cause medical costs were observed when the delay in initiating UMEC/VI was minimized. A reduced risk of a first severe exacerbation was also observed in these patients. After adjusting for baseline covariates and time-varying confounders using MSM, both COPD-related and all-cause medical costs during the year were found to be nearly 3% higher for each month that treatment initiation of UMEC/VI was delayed. This analysis also found a clear benefit of not delaying treatment with UMEC/VI, with a higher risk of a first severe exacerbation each month that treatment was delayed. These findings suggest that preventing a delay in treatment with UMEC/VI may result in lower medical costs than for similar patients who delay initiation. As exacerbations of COPD are an important cost driver [5], the observed economic benefit may be explained, in part, by the significantly lower risk of experiencing a severe exacerbation following initiation of UMEC/VI. However, additional research is needed to confirm this hypothesis. Consistent with the reduction in COPD-related healthcare costs reported here, several other studies support the economic benefit of UMEC/VI treatment initiation when compared with tiotropium monotherapy [25–28], open dual LAMA + LABA treatment [28], or no long-acting bronchodilator treatment [28]. However, whether a delay in initiation of UMEC/VI has further cost implications in these scenarios remains to be seen.

The results of this study suggest that healthcare providers may wish to consider the initiation of UMEC/VI earlier in the course of the disease. In the 2017 GOLD report the preferred first initiation of maintenance therapy is based on symptoms and exacerbation risk at presentation. In patients with particularly high symptom burden or who are symptomatic with a history of multiple exacerbations, initial LAMA + LABA therapy is recommended (GOLD Group B/D), while escalation from bronchodilator monotherapy to LAMA/LABA therapy is recommended for patients with a lower exacerbation risk but still experiencing persistent symptoms or those with a lower symptom burden who experience further exacerbations.

This study has demonstrated that reducing the delay in UMEC/VI initiation results in a reduced risk of severe exacerbations. Severe breathlessness, severe lung function impairment or a prior severe exacerbation are important predictors of future exacerbations [29–31], therefore it is possible that patients with earlier escalation to UMEC/VI will not only benefit in the short-term but may also have improved long-term outcomes by reducing or delaying exacerbation.

Observational data have been used to examine the potential benefits of initiating ICS therapy along with regular inhaled bronchodilators in patients with COPD earlier in the course of their disease, rather than using a step-wise approach to intensification. Interestingly, earlier treatment with ICS/LABA reduced severe COPD exacerbations and COPD-related healthcare expenditures [32], supporting the idea that reducing any delay in initiating combination therapy for COPD may be beneficial. According to the 2017 GOLD report, ICS/LABA dual therapy may be considered as an alternative to LAMA/LABA dual therapy [1]. However, a number of studies comparing dual LAMA/LABA and dual ICS/LABA combinations have consistently demonstrated the benefits of the LAMA/LABA combination and support the use of LAMA/LABA over ICS/LABA dual therapy, especially in those patients with high symptom burden and low exacerbation risk [19, 33–36].

To the best of our knowledge, this is the first outcomes study to assess the benefits of early use of a LAMA/LABA in a real-world setting. In addition, the use of MSM analysis to adjust for differences in multiple measurable factors, including time-varying confounders, between cohorts provides for more robust adjusting for confounding.

Claims data are a rich source for examining real-world patterns of healthcare outcomes and for assessing patterns of medication use, and are able to capture information from all providers caring for a patient. However, there can be limitations due to the lack of information relating to clinical measures (e.g. spirometry data and factors relating to exacerbations such as oxygen saturation) and patient characteristics, such as socioeconomic status and frequency of tobacco use. Furthermore, a claim for a filled prescription does not necessarily indicate that a medication has been used or used correctly; and, although a diagnosis code is included on the claim, it may not constitute evidence of the disease due to incorrect coding. The inability to measure and control for unobservable factors using administrative claims data will impact the interpretation of findings in this and similar studies. The population in this study included patients with commercial or MAPD coverage with a minimum of 18 months of continuous enrolment, the generalizability of results to other populations may be limited. As similar analyses using MSM have not been performed for other COPD medication classes (e.g. LAMA or ICS/LABA) we are not able to conclude whether these results are applicable to only UMEC/VI or are a consequence of a delay in initiation in general.

## Conclusions

This study demonstrates the economic and clinical benefits of preventing a delay in initiation of UMEC/VI dual therapy, with earlier initiation resulting in lower subsequent all-cause and COPD-related medical costs after initiation. This economic benefit may be explained, in part, by the lower risk of severe COPD exacerbation when UMEC/VI initiation is not delayed; however, additional research is needed to confirm this hypothesis, and to assess the relative benefits of initiation of UMEC/VI as a first-line therapy compared with escalation to UMEC/VI from bronchodilator monotherapies.

## Additional files

Additional file 1: COPD-related medications. COPD-related treatments identified by pharmacy claims in the baseline period. (DOCX 27 kb)

Additional file 2: Independent variables in treatment selection weighting models for cost analyses. Description of independent variables in treatment selection weighting models for the cost analyses. (DOCX 17 kb)

Additional file 3: Cohort counts by month of UMEC/VI initiation (*N* = 2200). Additional figure illustrating the number of patients initiating UMEC/VI each month. (DOCX 100 kb)

Additional file 4: MSM sensitivity analyses for COPD-related medical costs. Sensitivity analyses conducted to test the robustness of findings to changes in model structure and assumptions. (DOCX 18 kb)

## Abbreviations

CI: Confidence interval
COPD: Chronic obstructive pulmonary disease
ED: Emergency department
FDA: Food and Drug Administration
GEE: Generalized estimating equation
GOLD: Global Initiative for Chronic Obstructive Lung Disease
HR: Hazard ratio
ICD-10-CM: International Classification of Disease, 10^th^ edition, clinical modification
ICD-9-CM: International Classification of Disease, 9^th^ edition, clinical modification
ICS: Inhaled corticosteroid
ID: Identification
LABA: Long-acting β_2_-agonist
LAMA: Long-acting muscarinic antagonist
MA: Medicare Advantage
MAPD: Medicare Advantage with part D
MSM: Marginal structural models
ORD: Optum Research Database
SD: Standard deviation
UMEC: Umeclidinium
US: United States
USD: US dollars
VI: Vilanterol
