# Supplementary material for: The effect of delaying initiation with umeclidinium/vilanterol in patients with COPD: an observational administrative claims database analysis using marginal structural models
Source: Multidiscip Respir Med. 2018 Oct 11;13:38. doi: 10.1186/s40248-018-0151-6 (PMC6180385; doi:10.1186/s40248-018-0151-6)
Supplement: Supplementary file 1 — COPD-related medications. COPD-related treatments identified by pharmacy claims in the baseline period. (DOCX 27 kb) [file 40248_2018_151_MOESM1_ESM.docx]

**Additional file 1. COPD-related medications**

| **Drug class** | **Subclass** | **Medication** |
| --- | --- | --- |
| Controller  medications | LAMA | Tiotropium  Aclidinium  Umeclidinium  Glycopyrronium |
|  | LAMA/LABA combination | Umeclidinium/vilanterol  Tiotropium/olodaterol  Glycopyrrolate/indacaterol |
|  | ICS | Beclomethasone  Betamethasone  Budesonide (both inhaler and nebulizer)  Ciclesonide  Dexamethasone (nebulizer)  Flunisolide  Fluticasone  Mometasone  Triamcinolone |
|  | LABA | Arformoterol  Formoterol  Indacaterol  Olodaterol  Salmeterol |
|  | ICS/LABA combination | Fluticasone/salmeterol  Budesonide/formoterol  Fluticasone/vilanterol  Mometasone/formoterol |
|  | Methylxanthines | Aminophylline  Dyphylline  Theophylline |
|  | Others | Roflumilast  Alpha-1 proteinase inhibitor |
| Rescue medications | SABA | Albuterol  Bitolterol  Isoetharine  Isoproterenol  Levalbuterol  Metaproterenol  Pirbuterol  Terbutaline |
|  | SAMA | Ipratropium |
|  | SAMA/SABA | Ipratropium/albuterol |
|  | OCS | Betamethasone  Cortisone  Dexamethasone  Hydrocortisone  Methylprednisolone  Prednisolone  Prednisone  Triamcinolone |

COPD, chronic obstructive pulmonary disease; ICS, inhaled corticosteroid; LABA, long-acting β_2_-agonist; LAMA, long-acting muscarinic antagonist; OCS, oral corticosteroids; SABA, short-acting β_2_-agonist; SAMA, short-acting muscarinic antagonist
