# Supplementary material for: The effect of delaying initiation with umeclidinium/vilanterol in patients with COPD: an observational administrative claims database analysis using marginal structural models
Source: Multidiscip Respir Med. 2018 Oct 11;13:38. doi: 10.1186/s40248-018-0151-6 (PMC6180385; doi:10.1186/s40248-018-0151-6)
Supplement: Supplementary file 2 — Independent variables in treatment selection weighting models for cost analyses. Description of independent variables in treatment selection weighting models for the cost analyses. (DOCX 17 kb) [file 40248_2018_151_MOESM2_ESM.docx]

**Additional file 2. Independent variables in treatment selection weighting models for cost analyses**

Treatment selection weighting models for the cost analyses contained potential confounders as independent variables, including time (as a continuous month variable), baseline variables: age (categorized as: 40–<60, 60–<70, 70–<80, ≥80 years), gender, geographic region, insurance type, moderate exacerbation starting in baseline period, severe exacerbation starting in baseline period, comorbidities (respiratory infection or asthma diagnosis), medications (≥1 fill for short-acting β_2_-agonist [SABA], short-acting muscarinic antagonist [SAMA], SABA/SAMA, inhaled corticosteroid [ICS], long-acting β_2_-agonist [LABA], ICS/LABA, long-acting muscarinic antagonist [LAMA], methylxanthine, phosphodiesterase 4 [PDE-4] inhibitor, combination medication (ICS/LABA, LAMA/LABA, or SABA/SAMA), or COPD-recommended antibiotic; number of COPD controller regimen changes (categorized as 0, 1, 2+) and resource utilization (emergency department [ED] visit, inpatient [IP] visit, IP visit discharge, ambulatory visit count [adjusted for index visit in Month 1]), IP days); and monthly time-varying covariates: exacerbations (moderate exacerbation starting in the prior month, moderate exacerbation starting in the current month, severe exacerbation starting [current month; prior month]); comorbidities (respiratory infection or new asthma diagnosis in the current month, emphysema diagnosis in the prior 6 months [i.e. 6-month period ending in the prior month], Charlson comorbidity index [cumulative from baseline to the current month and prior month], COPD severity status; medications (≥1 fill for SABA, SAMA, or SABA/SAMA in the current month or prior month; number of SABA units, number of LAMA fills, number of oral corticosteroid fills, number of COPD-recommended antibiotic fills or ≥1 fill for ICS in the prior 6 months; ≥1 fill for ICS, LABA, ICS/LABA, LAMA, methylxanthine, PDE-4 inhibitor, combination medication, COPD-recommended antibiotic, or any COPD controller regimen change in the prior month); and resource utilization (ED visit, IP visit, IP visit discharge, and adjusted ambulatory visit count [current month; prior month]; index date-like visit and index date-like visit with a specialist [prior month]; and number of spirometry testing claims [prior 6 months]). Weighting models for the exacerbation analysis included the same covariates as the weighting models for the cost analyses, with the exception of the time-varying current month severe exacerbation start covariate.
