# Supplementary material for: The effect of delaying initiation with umeclidinium/vilanterol in patients with COPD: an observational administrative claims database analysis using marginal structural models
Source: Multidiscip Respir Med. 2018 Oct 11;13:38. doi: 10.1186/s40248-018-0151-6 (PMC6180385; doi:10.1186/s40248-018-0151-6)
Supplement: Supplementary file 3 — Cohort counts by month of UMEC/VI initiation (N = 2200). Additional figure illustrating the number of patients initiating UMEC/VI each month. (DOCX 100 kb) [file 40248_2018_151_MOESM3_ESM.docx]

**Additional file 3. Cohort counts by month of UMEC/VI initiation (N=2200)**

UMEC/VI, umeclidinium/vilanterol
