# Supplementary material for: The effect of delaying initiation with umeclidinium/vilanterol in patients with COPD: an observational administrative claims database analysis using marginal structural models
Source: Multidiscip Respir Med. 2018 Oct 11;13:38. doi: 10.1186/s40248-018-0151-6 (PMC6180385; doi:10.1186/s40248-018-0151-6)
Supplement: Supplementary file 4 — MSM sensitivity analyses for COPD-related medical costs. Sensitivity analyses conducted to test the robustness of findings to changes in model structure and assumptions. (DOCX 18 kb) [file 40248_2018_151_MOESM4_ESM.docx]

**Additional file 4. MSM sensitivity analyses for COPD-related medical costs**

| **Model** | **Cost ratio per 1 month change in treatment start** | **95% CI** | **p-value** | **Definition of treatment month exposure** |
| --- | --- | --- | --- | --- |
| **COPD-related medical cost outcome** |  |  |  |  |
| Final model:  Treatment month exposure is continuous  Ambulatory count is adjusted for index visit in Month 1  Weights truncated at 99.9^th^ percentile | 1.029 | 1.001–1.059 | 0.044 | Continuous |
| Final model with weights truncated at 99^th^ percentile | 1.031 | 1.002–1.060 | 0.035 | Continuous |
| Final model with weights not truncated | 1.025 | 0.997–1.055 | 0.078 | Continuous |
| Final model with ambulatory visit count unadjusted for index visit in Month 1 | 1.026 | 0.997–1.056 | 0.076 | Continuous |
| Final model with current month variables excluded | 1.025 | 0.995–1.056 | 0.100 | Continuous |
| Final model excluding current month variables except for current month ambulatory visit | 1.039 | 1.009–1.069 | 0.010 | Continuous |
| Final model with the addition of current month flags for ICS, LABA, LAMA, methylxanthine, and PDE-4 inhibitor | 1.032 | 1.001–1.065 | 0.043 | Continuous |
| Final model with treatment month exposure defined as binary (treatment starting in Months 7–12, compared with start in Months 1–6) | 1.143* | 0.924–1.412 | 0.217 | Binary (first/second half of year) |

*Cost ratio of second half of the year versus the first half of the year. CI, confidence interval; ICS, inhaled corticosteroid; LABA, long-acting β_2_-agonist; LAMA, long-acting muscarinic antagonist; PDE-4, phosphodiesterase-type 4
